# The Unrepaired Tetralogy of Fallot: A Tale of Delayed Presentation and Limited Access to Care

**DOI:** 10.7759/cureus.52407

**Published:** 2024-01-16

**Authors:** Pramod Bhattarai, Monika Karki

**Affiliations:** 1 Pulmonary Medicine, Howard University Hospital, Georgia Avenue, USA; 2 Critical Care Medicine, Larkin Community Hospital Palm Springs Campus, Hialeah, USA; 3 Internal Medicine, Harlem Hospital Center, New York, USA; 4 Internal Medicine, The Brooklyn Hospital Center, Brooklyn, USA

**Keywords:** cyanosis, overriding aorta, ventricular septal defect (vsd), eisenmenger syndrome, adult congenital heart disease (achd), tof:tetralogy of fallot

## Abstract

Tetralogy of Fallot (TOF) is a common cyanotic congenital heart disease characterized by four distinct anatomical features. While surgical repair has significantly improved long-term outcomes, some individuals, particularly those from low socioeconomic backgrounds who lack access to medical care, may suffer from complications such as pulmonary hypertension (pHTN) and heart failure. We present a case report of a young female with unrepaired TOF who presented with acute-on-chronic hypoxic respiratory failure and heart failure, highlighting the complex nature and challenges associated with this condition.

## Introduction

This article was previously presented as a poster at the 2023 CHEST Annual Scientific Meeting on October 9, 2023.

TOF is a cyanotic congenital heart disease characterized by four anatomical features, as described by Dr. Fallot in 1888: overriding aorta, pulmonary infundibular stenosis, ventricular septal defect (VSD), and right ventricular hypertrophy [1]. It accounts for approximately 7%-10% of infants with congenital heart disease (CHD) and affects about one in 3500 live births [2]. The etiology is multifactorial, with a familial risk estimated at 3% [3]. The presentation and clinical state depend on the degree of right ventricular outflow tract obstruction, ranging from asymptomatic cases with minimal obstruction to those with significant cyanosis in moderate to severe obstruction [4].

## Case presentation

**Figure 1:**
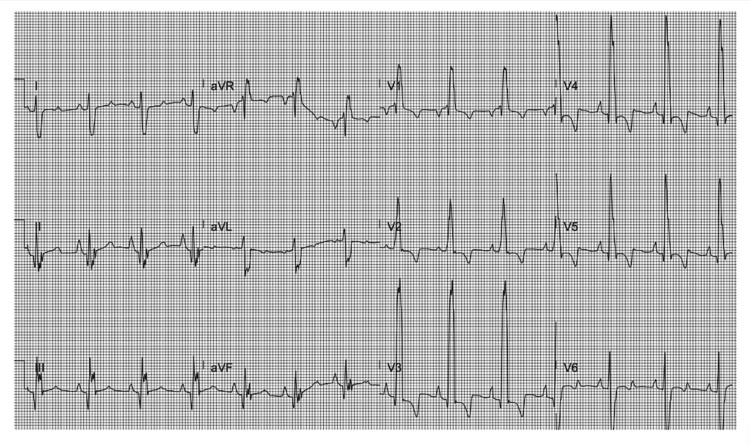
EKG showing normal sinus rhythm, bi-atrial enlargement, right bundle branch block, and left posterior fascicular block

**Figure 2:**
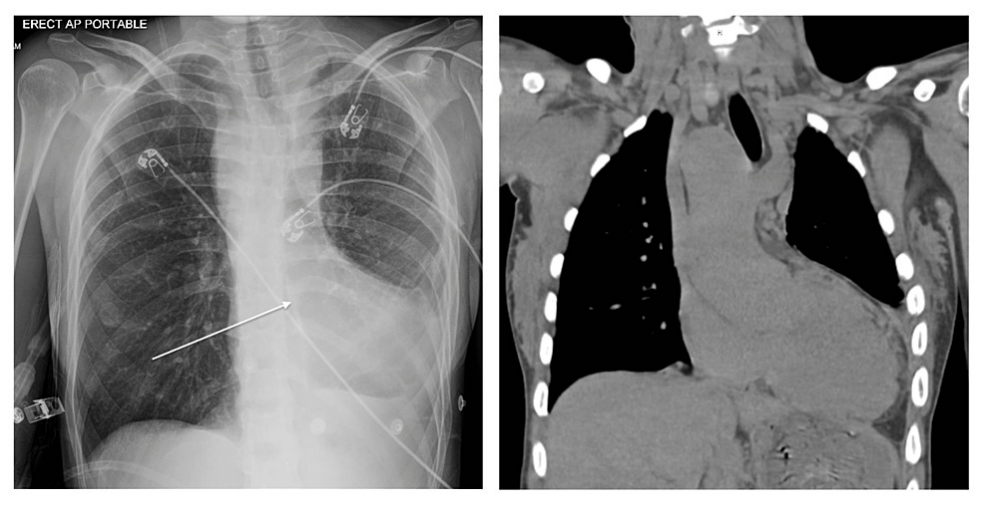
CXR and CT chest showing boot-shaped heart

**Figure 3:**
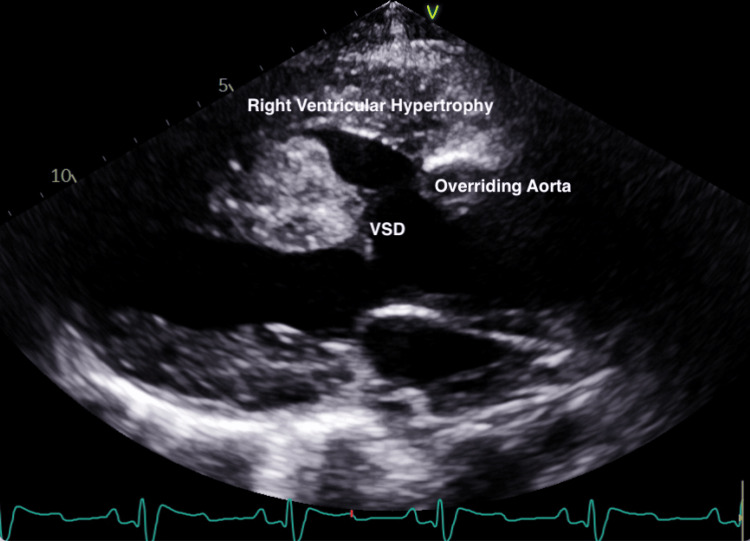
Echocardiogram showing VSD, overriding aorta, and right ventricular hypertrophy

**Figure 4:**
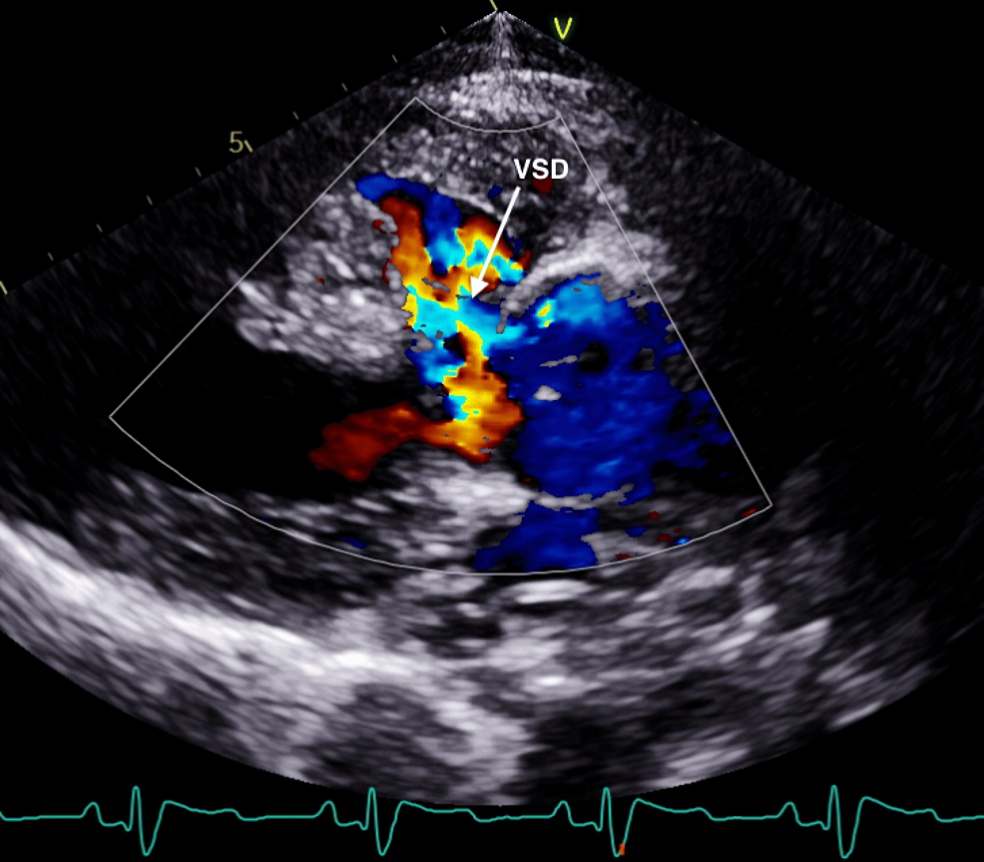
Echocardiogram with color doppler showing right-to-left shunt VSD

A 31-year-old Cuban female with a history of unrepaired TOF, pHTN, and secondary polycythemia presented with shortness of breath after running out of oxygen following her recent immigration to the USA. Physical examination revealed hypoxia, with an initial oxygen saturation of 75% that improved to 90% on high-flow oxygen. A grade 2/6 pan-systolic murmur, displaced point of maximal impulse, and clubbed fingers were also noted. An electrocardiogram showed normal sinus rhythm, bi-atrial enlargement, a right bundle branch block, and a left posterior fascicular block (Figure 1). The chest X-ray demonstrated an enlarged “boot-shaped” heart (Figure 2). Coronary tomography of the chest revealed aneurysmal dilatation of the ascending thoracic aorta measuring up to 5.8 cm. An echocardiogram confirmed the diagnosis of TOF, showing an enlarged overriding aorta, right ventricular outflow tract obstruction, right ventricular hypertrophy, and large peri-membranous ventricular septal defect (Figures 3, 4), with a right-to-left shunt consistent with Eisenmenger syndrome. Pulmonary artery pressure was estimated to be 55-60 mm Hg based on the echocardiogram findings, and there was dilation of the aortic root and ascending aorta with mild aortic regurgitation. Additionally, there was a significant reduction in left ventricular systolic function, with an ejection fraction of 28.8%. Laboratory tests indicated secondary polycythemia with an elevated hemoglobin of 20.2 and a hematocrit (HCT) level of 62.8. She was initiated on oxygen therapy and sildenafil, and a referral was made for surgical intervention at a tertiary heart center. However, she declined the surgical intervention and opted to continue medical management with spironolactone, valsartan, sildenafil, and oxygen as needed.

## Discussion

Over the years, advances in medical interventions and surgical techniques have greatly improved the survival rates of patients with CHD, including TOF. In the 1960s, the survival rate for individuals with CHD reaching adulthood was only 15%, but it had increased to 85%, with the number of adults with CHD in North America having almost surpassed one million in 2016 [5]. Despite these improvements, the survival rate for adult CHD patients beyond their third and fourth decades remains lower compared to the general population [3]. Studies have shown that individuals with unrepaired TOF who survive into adulthood beyond their thirties tend to have milder pulmonary obstruction, left ventricular hypertrophy, and extracardiac shunts such as patent ductus arteriosus or systemic-to-pulmonary artery shunting [6,7]. However, even with these favorable factors, the mortality rate remains high, with only 24% of individuals surviving beyond 10 years [4]. The long-term survival rates for unrepaired TOF patients beyond 20 years are as low as 10%, with only 3% surviving beyond 40 years, compared to a 90% survival rate among those who have undergone surgical repair [1,3].

The Blalock-Taussig procedure is a staged palliation procedure for TOF that has been performed since 1954 [1]. Early surgical repair is generally preferred as it minimizes the duration of exposure to prolonged hypoxemia and cyanotic spells, thereby potentially preserving myocardial mechanical and electrical functions [8]. However, even for patients who did not undergo surgical correction of TOF at a younger age, total surgical correction can still be considered in individuals aged 40 years old or older, as the operative risk is around 3%, and long-term survival has shown improvement [9].

In our patient’s case, surgical repair was considered as a potential treatment option. Arrangements were made for her transfer to a tertiary heart center equipped with adult congenital cardiologists and transplant capabilities, offering the possibility of repair or a heart-lung transplant to address her Eisenmenger syndrome, as individuals with VSD and Eisenmenger syndrome have the best prognosis with a heart-lung transplant [10]. However, the patient declined the transfer and surgical intervention due to financial concerns and personal reasons, as she was not from the United States. Subsequently, she was managed medically. Considering her secondary polycythemia, a phlebotomy option was evaluated; however, based on her HCT level of 62.8%, phlebotomy was not performed. Current guidelines do not recommend prophylactic phlebotomy in secondary polycythemia patients unless the HCT level exceeds 65% or signs and symptoms of hyperviscosity, thrombosis, or non-thrombotic complications are present [11].

Specific guidelines for managing acute heart failure in ACHD patients are lacking. In patients with pHTN and/or persistent cardiac shunts, maintaining a balance between pulmonary vascular resistance (PVR) and systemic vascular resistance (SVR) is crucial. Increasing PVR can lead to decreased cardiac output, while decreasing SVR can increase the risk of right-to-left shunting and systemic desaturation [5]. Therefore, it is important to avoid triggers such as hypoxemia, high HCT, and hypercapnia, which can increase PVR [5]. Similarly, the use of vasodilators and general anesthesia should be approached with caution, as they can reduce SVR [5]. In cases of persistent right-to-left shunting, the use of intravenous lines with attached bubble filters is recommended to minimize the risk of paradoxical air or thromboembolism [5]. Regular monitoring of coagulation factors and platelets is essential in cyanotic patients with secondary erythrocytosis, as they are at increased risk of bleeding and thrombosis. Although there are no specific guidelines for heart failure management in ACHD patients, the use of diuretics, renin-angiotensin-aldosterone system inhibitors, beta-blockers, and mineralocorticoid receptor antagonists is generally recommended to alleviate symptoms and improve prognosis [5,12]. In our patient’s case, her management plan included the continuation of her home medications, with sildenafil for pHTN and right ventricular failure secondary to pHTN, as well as spironolactone and valsartan for heart failure with reduced ejection fraction. Additionally, oxygen therapy was provided as needed to optimize her oxygen levels and prevent hypoxemia.

## Conclusions

Due to increasing immigration from developing countries, we encounter a growing number of patients with unrepaired TOF and its associated complications. This case serves as an example of the significant comorbidities and long-term complications that individuals with unrepaired TOF who have survived into adulthood may experience due to prolonged cyanosis and polycythemia resulting from limited access to adequate medical care. Currently, there are no established guidelines for managing these conditions; therefore, management should be individualized based on each patient’s condition and comorbidities. It is important to recognize that HCT levels up to 65 may not require phlebotomy unless the patient exhibits symptoms. Similarly, an oxygen requirement between 75% and 85% is generally acceptable in this patient population. Ultimately, for patients like ours, a lung and heart transplant could be an effective approach to managing their condition and ensuring long-term survival. These interventions can provide comprehensive treatment by addressing the underlying cardiac abnormalities and associated complications, offering the best chance for improved quality of life and extended survival. Considering the increasing prevalence of patients with unrepaired TOF and the complexity of managing their long-term complications, further research and the development of specific guidelines are warranted.

This case report adds to the existing literature on unrepaired congenital heart diseases presenting with complications in adulthood. Although not unique, it does add to the existing body of evidence and further recognizes the need for focused ACHD care. 
